# Genome Sequence of Bacillus megaterium Strain YC4-R4, a Plant Growth-Promoting Rhizobacterium Isolated from a High-Salinity Environment

**DOI:** 10.1128/genomeA.00527-18

**Published:** 2018-06-21

**Authors:** Juan Ignacio Vílchez, Qiming Tang, Richa Kaushal, Wei Wang, Suhui Lv, Danxia He, Zhaoqing Chu, Heng Zhang, Renyi Liu, Huiming Zhang

**Affiliations:** a Shanghai Center for Plant Stress Biology and CAS Center for Excellence in Molecular Plant Sciences, Chinese Academy of Sciences, Shanghai, China; b University of Chinese Academy of Sciences, Beijing, China; c Shanghai Key Laboratory of Plant Functional Genomics and Resources, Shanghai Chenshan Plant Science Research Center, Shanghai, China; d Center for Agroforestry Mega Data Science and FAFU-UCR Joint Center for Horticultural Biology and Metabolomics, Haixia Institute of Science and Technology, Fujian Agriculture and Forestry University, Fuzhou, China

## Abstract

Here, we report the complete genome sequence for Bacillus megaterium strain YC4-R4, a highly salt-tolerant rhizobacterium that promotes growth in plants. The sequencing process was performed by combining pyrosequencing and single-molecule sequencing techniques. The complete genome is estimated to be approximately 5.44 Mb, containing a total of 5,673 predicted protein-coding DNA sequences (CDSs).

## GENOME ANNOUNCEMENT

Bacillus megaterium strain YC4-R4 is a highly salt-tolerant Gram-positive bacterium which was isolated from rhizospheric soil of a Spartina anglica plant at Zhangpu Yanchang in Fujian Province, China. This rhizobacterial collection was enriched by *Bacillaceae* family members that can produce spores, probably reflecting environmental selection for microbes can survive under high-salinity conditions (1, 2). In addition, most of the isolated strains were capable of producing one or multiple effectors, including auxins, aminocyclopropane-1-carboxylate deaminase (ACCd), polyamines, and metabolites that help increase solubility of phosphate, displaying the characteristics commonly reported in plant growth-promoting bacteria (3–8). We have characterized B. megaterium YC4-R4 as a phosphorous solubilizer and siderophore producer. Importantly, we have observed a strong plant growth-promoting effect, as well as a moderate enhancement of plant tolerance to drought stress when B. megaterium YC4-R4 is used as an inoculant (Juan Ignacio Vílchez and Huiming Zhang, unpublished data). This strain has been deposited in the China General Microbiological Culture Collection Center (CGMCC) collection with the reference number 14421.

We sequenced the complete genome of B. megaterium YC4-R4 by a combination of pyrosequencing and single-molecule sequencing techniques. The pyrosequencing was performed with an Illumina HiSeq platform (Core Facility of Genomics, Shanghai Center for Plant Stress Biology, China), and the single-molecule sequencing was performed with a PacBio platform (Tianjin Biochip Corporation, China) (9–12). The shotgun sequencing strategy was applied to the pyrosequencing, and 12,257,603 paired reads (150 bp) were obtained, with a sequencing depth of approximately 252-fold. A total of 6 plasmids between 9 and 162 kbp were sequenced, together with 5,129 kbp of chromosomal genes. Meanwhile, the single-molecule sequencing produced 85,919 reads, with a mean read length of 11,221 bp and *N*_50_ length of 16,259 bp. The total number of sequenced bases was 961,774,920. For *de novo* assembly, CANU version 1.5 was used with default parameters; the genome correction step was performed by using Illumina data with the support of the Pilon version 1.18 software (13, 14). This assembly yielded an average of 906,920 bp. The genome is assembled completely by the 6 contigs and not by scaffolds. Subsequently, the estimated genome size of 5.44 Mb was deduced from the contigs. Genes including protein-coding DNA sequences (CDSs) were predicted by a pipeline implemented by Prokka version 1.12 (15). On a whole-genome scale, the G+C content accounts for only 38.38% of the B. megaterium YC4-R4 genome, which was found to contain 5,673 protein-coding genes, 5 rRNA operons, and 130 tRNA genes.

With the annotated chromosomal genome sequence of B. megaterium YC4-R4, many biological pathways can be predicted, such as those related to flagella, spores, and polysaccharides. The genome annotation also provides genetic information about the biosynthesis of xeroprotectants, antioxidants, lipopolysaccharides, outer membrane adhesins, cell wall-binding protein, and extracellular receptors, proteins related to the degradation of toxic compounds. Further investigation will provide valuable insights into the molecular mechanisms underlying plant growth promotion induced by B. megaterium YC4-R4, as well as facilitate other biotechnologically based applications.

### Accession number(s).

The complete genome sequence of B. megaterium YC4-R4 has been deposited in the TBL/EMBL/GenBank databases under the BioProject number PRJNA430758 and accession numbers CP026736 to CP026741.
